# Classification of Multiple Sclerosis Clinical Profiles via Graph Convolutional Neural Networks

**DOI:** 10.3389/fnins.2019.00594

**Published:** 2019-06-12

**Authors:** Aldo Marzullo, Gabriel Kocevar, Claudio Stamile, Françoise Durand-Dubief, Giorgio Terracina, Francesco Calimeri, Dominique Sappey-Marinier

**Affiliations:** ^1^CREATIS, CNRS UMR5220, INSERM U1206, Université de Lyon, Université Lyon 1, INSA-Lyon, Villeurbanne, France; ^2^Department of Mathematics and Computer Science, University of Calabria, Rende, Italy; ^3^Service de Neurologie A, Hôpital Neurologique, Hospices Civils de Lyon, Lyon, France; ^4^CERMEP–Imagerie du Vivant, Université de Lyon, Lyon, France

**Keywords:** multiple sclerosis, graph neural networks, graph-derived metrics, diffusion tensor imaging, connectome

## Abstract

Recent advances in image acquisition and processing techniques, along with the success of novel deep learning architectures, have given the opportunity to develop innovative algorithms capable to provide a better characterization of neurological related diseases. In this work, we introduce a neural network based approach to classify Multiple Sclerosis (MS) patients into four clinical profiles. Starting from their structural connectivity information, obtained by diffusion tensor imaging and represented as a graph, we evaluate the classification performances using unweighted and weighted connectivity matrices. Furthermore, we investigate the role of graph-based features for a better characterization and classification of the pathology. Ninety MS patients (12 clinically isolated syndrome, 30 relapsing-remitting, 28 secondary-progressive, and 20 primary-progressive) along with 24 healthy controls, were considered in this study. This work shows the great performances achieved by neural networks methods in the classification of the clinical profiles. Furthermore, it shows local graph metrics do not improve the classification results suggesting that the latent features created by the neural network in its layers have a much important informative content. Finally, we observe that graph weights representation of brain connections preserve important information to discriminate between clinical forms.

## 1. Introduction

Multiple sclerosis (MS) is the most frequent disabling neurological disease in young adults. While its etiology remains unknown, MS is a chronic disease of the central nervous system, characterized by inflammation, demyelination, and neurodegenerative pathological processes (Polman et al., 2011). In 85% of the patients, disease onset is characterized by a first acute clinical episode [called clinically isolated syndrome (CIS)], including optic neuritis, paresthesia, paresis, and fatigue (McDonald et al., 2001), evolving into a relapsing-remitting (RRMS) course, and after a delay varying between 15 and 20 years, into a secondary-progressive (SPMS) course, leading to long-term disability. The remaining 15% of MS patients starts with the primary-progressive course (PPMS) (Miller et al., 2005a,b). A revised version of this classification of the MS clinical courses has been proposed in 2014 by Lublin et al. (2014). In this revision, two main forms of the disease are considered: the relapsing remitting MS (CIS and RRMS patients) and the progressive MS (SPMS and PPMS patients), each one being wether active or not active.

The course of the disease and the risk for developing permanent disability are very different from one patient to another and the prediction of long-term disability is still not possible in a new MS patient. Today's neurologist challenge is to predict the individual patient evolution and response to therapy based on the clinical, biological, and imaging markers available from disease onset. Long-term clinical studies have been conducted to determine the clinical predictors of disability accumulation in MS (Degenhardt et al., 2009; Soldán et al., 2015). In RRMS and SPMS, several negative prognostic factors were identified such as the onset of progression, higher early relapse rate, greater disability in the first 5 years, and shorter interval to the second relapse. However, none of these predictors are available at the beginning of the disease (Confavreux et al., 2003; Confavreux and Vukusic, 2006; Scalfari et al., 2010).

Among the available information with a potential prognostic value at the CIS stage, MRI remains the most promising. A lot of efforts have been concentrated on the identification and characterization of MS lesions (Brosch et al., 2015; Valverde et al., 2017). While conventional T2 lesion load is moderately correlated with the patient clinical status, it can predict the increase of disability scores, such as the Expanded Disability Status Scale (EDSS) and the Multiple Sclerosis Functional Composite (MSFC) (Barkhof, 2002). Global brain atrophy constitutes a potential marker, as it even exists at the early stages of MS. However, its predictive value is still controversial, probably due to its methodological limitations (Durand-Dubief et al., 2012). Measurement of subcortical gray matter atrophy could be of special interest if appropriate tools were available in clinic (Hannoun et al., 2012). Indeed, atrophy in the thalamus was recently reported to be an early marker of the neurodegeneration processes occurring throughout the disease progression (Azevedo et al., 2018). Regional atrophy in the whole brain was also studied, showing a specific pattern of the atrophy progression within the central nervous system, starting in the posterior cingulate cortex before spreading in the whole cortex (Eshaghi et al., 2018). More advanced MRI techniques, such as brain volumetry, magnetization transfer imaging (MTI) and diffusion-tensor imaging (DTI) are promising tools in that perspective (Rovira et al., 2013). Reflecting more specifically the demyelination and remyelination processes, have been shown to predict deterioration of cognitive functions in patients with early MS stages followed during 7 years (Deloire et al., 2011). However, these advanced techniques are not always available in clinical routine. In contrast, DTI becomes more available in clinical environment and provides an effective mean for the quantification of demyelination and axonal loss in CIS patients (Sbardella et al., 2013). Furthermore, it has recently been shown that diffusivity measurements in CIS patient's cerebellar white matter (decreased fractional anisotropy) can be predictive of a shorter conversion into a clinically definite MS (Kugler and Deppe, 2018). Therefore, we propose in this work a new approach for the automatic classification of MS clinical profiles based on brain DTI acquisition.

MRI data are usually represented as images. However, new data representation approaches were developed based on graph theory. Recently applied in neurosciences, graph-based models opened new perspectives for the exploration of brain structural and functional connectivity by means of graph-derived metrics (Rubinov and Sporns, 2010). In this context, few machine leaning approaches have been developed for the classification of MS clinical forms. Stamile et al. (2015) applied Support Vector Machines (SVM) to graph-based representation of the brain for the classification of MS patients clinical courses. In particular, Brain structural connectivity graphs were extracted from DTI data and several experiments were performed to classify RR vs. PP, RR vs. SP, PP vs. SP, and RR vs. PP vs. SP clinical profiles. Both weighted and binary graphs have been considered and the best performances were obtained with unweighted graphs for most of the classification tasks. In Kocevar et al. (2016), a similar strategy has been used. Six global features (graph density, assortativity, transitivity, global efficiency, modularity, and characteristic path length) were extracted from the structural connectivity graphs to enhance the performance of the SVM classification of MS clinical profiles. High level of accuracy were obtained in the HC vs. CIS, CIS vs. RR, RR vs. PP, RR vs. SP, SP vs. PP, and CIS vs. RR vs. SP tasks. This work demonstrated the better sensitivity of the modularity and assortativity metrics to achieve the best performances. These approaches provided remarkable results on binary classification tasks but were unable to classify the four possible MS profiles at once.

More recently, Neural Networks (NN) based approaches showed promising results for the analysis and classification of images in a wide range of applications (Goodfellow et al., 2016).

More specifically in the context of MRI analysis in MS, Whang et al. exploited complex CNN to differentiate MS patients from healthy controls with an accuracy greater than 98% based on T1-weighted MRI (Wang et al., 2018). The same task was addressed by Maleki et al. and Zhang et al. where CNN achieved similar results (Maleki et al., 2012; Zhang et al., 2018). In Ion-Mărgineanu et al. (2017) the authors demonstrated the potential of using simple CNN to classify MS clinical courses. In particular, they exploited features extracted from magnetic resonance spectroscopic images combined with brain tissue segmentations of gray matter, white matter, and lesions.

Since the first definition of Graph Neural Networks (GNN) (Scarselli et al., 2009), a huge effort was made to extend neural networks with the purpose of processing graph structures data. By implementing a function that maps a graph and its nodes into an m-dimensional Euclidean space, the GNN model can directly process many types of graphs (e.g., acyclic, cyclic, directed, and undirected). An extension of this approach was proposed by Kipf and Welling (2016), which introduced a Graph Convolutional Neural Network (GCNN) model that is able to achieve promising results by properly managing structured data and capturing hidden information from graphs.

In this work, we used the GCNN model to classify MS patients into four clinical profiles [CIS, RR, SP, PP] using the graph structural connectivity information. Beside the use of brain connectivity graphs directly as an input to the NN, we also investigate the potential role of graph local features in further improving classification performances. Finally, we perform our experiments using both weighted and unweighted connectivity matrices of the brain structure, in order to understand the role played by edge weights in the classification process.

## 2. Materials and Methods

Our method is divided in three steps: (i) structural connectivity information is extracted from the images in order to produce a graph representation of the MRI; (ii) a feature matrix is extracted from each graph and local graph metrics are computed; (iii) the adjacency matrix, together with the local graph features matrix, is used as input for the GCNN to perform the classification task.

In the following, we illustrate how structural connectivity information are extracted from the images in order to produce a graph representation of the MRI. Then, we describe the NN architecture used for the classification task. Finally, we provide a description of the graph features considered in this work.

### 2.1. MRI Acquisition and Data Set Description

The MS population consisted of 12 CIS, 30 RR, 28 SP, 20 PP examined longitudinally every 6 months during 3 years and then every year during 4 more years. A total of 580 exams were processed for classification. In addition, 24 healthy controls (HC) subjects, age, and sex matched with the MS patients, were considered in the experiments. This prospective study was approved by the local ethics committee (CPP Sud-Est IV) and the French national agency for medicine and health products safety (ANSM). Written informed consents were obtained from all patients prior to study initiation. A description of clinical data is reported in Table 1. Diagnosis and MS profile were established according to the McDonald criteria (McDonald et al., 2001; Lublin et al., 2014), while disability was assessed with Extended Disability Status Scale (EDSS).

**Table 1:** Information on the data set for the different clinical profiles (HC,CIS, RR, SP, PP).

	**HC**	**CIS**	**RR**	**SP**	**PP**
Number of patients (%Male/Female)	24 (42/57)	12 (50/50)	30 (20/80)	28 (61/39)	20 (45/55)
Age at first scan (years)	35.7 (10.1)	30.88 (6.4)	27.57 (7.8)	27.64 (7.6)	34.99 (6.1)
Disease duration (years)	–	1.50 (1.54)	6.75 (4.81)	13.12 (5.84)	5.90 (2.60)
EDSS median (range)	–	0.5 (0–4)	2.0 (0–4.5)	5.0 (3–7)	4.0 (2.5–6.5)
Total number of scans	24	63	190	199	126

MR examinations were performed on a 1.5T Siemens Sonata system (Siemens Medical Solution, Erlangen, Germany) using an 8-channel head-coil. The MR protocol consisted in the acquisition of a sagittal 3D-T1 sequence (1 × 1 × 1 *mm*^3^, TE/TR = 4/2000 ms) and an axial 2D-spin-echo DTI sequence (TE/TR = 86/6900 ms; 2 × 24 directions of gradient diffusion; *b* = 1000 *s*.*mm*^−2^, spatial resolution of 2.5 × 2.5 × 2.5 *mm*^3^) oriented in the AC-PC plane.

### 2.2. Brain Structural Connectivity Graph

As previously described by Kocevar et al. (2016), the data processing for the extraction of brain structural connectivity is composed of three steps:

1. First, each voxel of the T1-weighted MR images is labeled in four classes, depending on the corresponding tissue type [white matter (WM), cortical GM, sub-cortical GM, cerebro-spinal fluid (CSF)]. In order to perform the classification a segmentation of the Cortical and sub-cortical parcellation using FreeSurfer (Fischl et al., 2004) is performed on the T1 images. The segmentation is also used to define the graph nodes (q = 84).
2. Second, the diffusion images are pre-processed by applying correction of Eddy-current distortions (Jenkinson et al., 2012) and skull stripping.
3. Third, MRtrix spherical deconvolution algorithm (Tournier et al., 2012) is used to estimate main diffusion directions in each voxel of diffusion images. Starting from the previous tissue-class labeling, a probabilistic streamline tractography algorithm is applied to generate fiber-tracks in voxels labeled as WM voxels. Symmetrical connectivity matrix $\text{A}\in {\mathbb{N}}_{+}^{q\times q}$ is then generated for each subject through the combination of GM segmentation and WM tractography.

In detail, let Ψ : ${\mathbb{N}}_{1}^{2}\to \mathbb{N}$ be the number of fibers connecting two nodes *i* and *j*. Then, each element of the connectivity matrix *A* is *a*_*i, j*_ = Ψ(*i, j*). In particular, *A* represents the adjacency matrix of the weighted undirected graph *G* = (*V, E*, ω) where *V* (|*V*| = *q*) is the set containing the segmented GM brain regions, *E* is the graph edges set defined as:

$$\begin{array}{l} E=\left\{\left\{i,j\right\}\;|\text{ Ψ}\left(i,j\right)>0\;\forall \;1\leq i,j\leq q\right\} \end{array}$$

Finally, a weighted undirected graph $G^{1}=\left(V^{1},E^{1},\omega \right)$ is created, starting from the undirected graph G=(V,E,ω) by applying the graph function $\Upsilon :\;G\;\to \;G^{1}$. The resulting graph contains only the strongly connected regions with respect to a given threshold τ ∈ ℝ_[0, 1]_. In particular, Υ performs the following mapping:

$$\begin{array}{l} \begin{array}{cc} V^{1}=V & E^{1}=L\left(1,\ldots ,T\right),T=\frac{\left(q^{2}-q\right)\tau }{2} \end{array} \end{array}$$

where *L* is the list of graph edges (*E*) sorted in ascending order of weight. This results in a weighted undirected brain connectivity graph that is used for this work.

### 2.3. Notation

For the description of our method, we introduce the following notations. We denote scalar values with small letters (e.g., *a*), 1-dimensional vectors with bold small letters (e.g., *a*), matrices with boldface capital letters (e.g., *A*) where **A**′ is the transpose of **A**. G=(V,E) is an undirected graph, where *V* is the set of vertex and *E* is the set of edges. For each vertex *v*∈*V* let **x** ∈ ℝ^*d*^ be the associated feature vector. If not differently specified, given a graph G=(V,E) we denote by *n* = |*V*| the number of nodes of the graph.

### 2.4. Graph-Based Neural Networks

Graph Convolutional Networks are neural network models that directly encode graph structure. Let **A** ∈ ℝ^|*V*| × |*V*|^ be the adjacency matrix of a graph G=(V,E) and **X** ∈ ℝ^|*V*| × *d*^ the feature matrix associated with it. The Graph Convolution layer with *k* output nodes is a function *H*:ℝ^|*V*| × *d*^ × ℝ^|*V*| × |*V*|^ → ℝ^|*V*| × *k*^, such that:

$$\begin{array}{l} H\left(\text{X},\text{A}\right):=\sigma \left(\hat{\text{A}}\text{XW}\right) \end{array}$$

where **W** ∈ ℝ^*d* × *M*^ is a weight matrix, σ is a non-linear activation function (e.g., ReLU) and $\hat{\text{A}}$ is the re-normalized adjacency matrix, i.e., $\hat{\text{A}}:={\tilde{\text{D}}}^{-1/2}\tilde{\text{A}}{\tilde{\text{D}}}^{-1/2}$ with $\tilde{\text{A}}:=\text{A}+{\text{I}}_{\left|V\right|}$ (the identity matrix) and $\tilde{\text{D}}$ is the diagonal node degree matrix. Roughly speaking, given an adjacency matrix **A** and a set of features **x** for each node (row of **A**), the GC layer convolves the neighborhood of every nodes to produce an embedding of these nodes.

The architecture proposed in this work is composed of one Graph Convolution layer (*k* = 100) with ReLU activation function followed by a Fully Connected Network with *softmax* activation to handle the multi-class classification problem. Dropout (α = 0.3) is used to reduce overfitting.

### 2.5. Graph Local Features

Feature extraction is an important task for graph classification. Indeed, while adjacency matrices represent exactly the structure of the graph, features encode latent patterns, or measure simple characteristics of graphs which could be useful for a better characterization and classification. Particularly, in the brain connectivity domain, several measures are able to detect functional integration and segregation to quantify centrality of individual brain regions or pathways, characterize patterns of local anatomical circuitry, and to test resilience of networks to insult (Rubinov and Sporns, 2010). These network measures have binary and weighted variants, where weighted variants of measures are typically generalizations of binary variants obtained by considering edge weights in the computation. In this work four local measures were identified^1^, according to the method described in Rubinov and Sporns (2010). Below, we report a detailed description of their weighted and unweighted version. Nevertheless, we refer the interested reader to Rubinov and Sporns (2010) for a complete description of the most commonly used measures of local and global connectivity, as well as their neurobiological interpretations.

#### 2.5.1. Node Degree

The degree of a node is the number of connections of that node. The weighted version of the metric (strength) also considers the weights of the edges into account. Let *N* be the set of all nodes in the network and *a*_*ij*_ the connection status between *i* and *j*, i.e., equals 1 if there is a link between these two nodes 0 otherwise. The degree of an unweighted graph can be calculated as follows:

$$\begin{array}{l} D_{i}=\underset{j\in N}{\sum }a_{ij} \end{array}$$

The weighted version of the metric (strength) also considers the weights of the edges into account. Let *w*_*ij*_ the connection weight between *i* and *j*, the weighted degree of a weighted undirected graph can be calculated as follows:

$$\begin{array}{l} D_{i}^{w}=\underset{j\in N}{\sum }w_{ij} \end{array}$$

#### 2.5.2. Clustering Coefficient

The clustering coefficient is the fraction of triangles around a node and is equivalent to the fraction of node's neighbors that are neighbors of each other. Let *t*_*i*_ be the number of triangles around a node *i* computed as follows:

$$\begin{array}{l} t_{i}=\frac{1}{2}\underset{j,h\in N}{\sum }a_{ij}a_{ih}a_{jh} \end{array}$$

The clustering coefficient per each node *i* is computed as:

$$\begin{array}{l} CC_{i}=\frac{2t_{i}}{k_{i}\left(k_{i}-1\right)} \end{array}$$

The weighed version of the clustering coefficient is obtained by replacing the number of triangles *t*_*i*_ with the sum of triangle intensities:

$$\begin{array}{l} CC_{i}^{w}=\frac{2}{k_{i}\left(k_{i}-1\right)}\underset{j,k}{\sum }{\left({\tilde{w}}_{ij}{\tilde{w}}_{jk}{\tilde{w}}_{ki}\right)}^{1/3} \end{array}$$

where weights are normalized by the largest weight in the network, ${\tilde{w}}_{ij}=w_{ij}/max\left(w_{ij}\right)$.

#### 2.5.3. Local Efficiency

Let first define the global efficiency as the average of inverse shortest path length. The local efficiency is the global efficiency computed on the neighborhood of the node, and is related to the clustering coefficient. It can be defined as follows:

$$\begin{array}{l} E_{i}=\frac{1}{n}\underset{i\in N}{\sum^{\text{​}}}\frac{\sum {}_{j,h\in N,j\neq i}a_{ij}a_{ih}{\left[d_{jh}(N_{i})\right]}^{-1}}{k_{i}\left(k_{i}-1\right)} \end{array}$$

where *E*_*i*_ is the local efficiency of node *i*, and *d*_*jh*_(*N*_*i*_) is the length of the shortest path between *j* and *h*, that contains only neighbors of *i*. By considering weights in the calculation, the formula can be extended to the weighted version as follows:

$$\begin{array}{l} E_{i}^{w}=\frac{1}{2}\underset{i\in N}{\sum^{\text{​}}}\frac{\sum {}_{j,h\in N,j\neq i}{\left(w_{ij}w_{ih}{\left[d_{jh}^{w}(N_{i})\right]}^{-1}\right)}^{1/3}}{k_{i}(k_{i}-1)} \end{array}$$

#### 2.5.4. Betweenness Centrality

Betweenness centrality is the fraction of all shortest paths in the network that pass through a given node. Nodes with high betweenness centrality are considered hub nodes and determine important regions in a network. In terms of brain networks this measure helps to detect important anatomical or functional connections. It is defined as follows:

$$BC_{i}=\frac{1}{\left(n-1\right)\left(n-2\right)}\underset{\begin{array}{c} h,j\in N\\ h\neq j,h\neq i,j\neq i \end{array}}{\sum }\frac{\rho_{hj}\left(i\right)}{\rho_{hj}}$$

where ρ_*hj*_ is the number of shortest paths between *h* and *j*, and ρ_*hj*_(*i*) is the number of shortest paths between *h* and *j* that pass through *i*. Betweenness centrality is equivalent on weighted networks, provided that path lengths are computed on respective weighted paths.

### 2.6. Statistical Analysis

To address the variability introduced by each patient, a linear mixed-effects model was applied separately to each local graph metric in each graph node:

(1) $$\begin{array}{r} Response_{ij}=\beta_{0}+\beta_{1}\left(Clinical\;phenotype_{i}\right)\\ +\beta_{2}\left(Scan\;Session_{ij}\right)+b_{0i}+\epsilon_{ij} \end{array}$$

In this model, the predicted response of interest for subject *i* at time *j* is determined by fixed effects, represented by β_1_ and β_2_. Subject-specific effects are represented by *b*_0*i*_, allowing a random interception per subject *i*.

The linear mixed-effects models were fitted using the “lme4" package in R (Bates et al., 2015) and the significance of the fixed effects and the interaction term is tested applying the Kenward-Roger approximation to estimate the degrees of freedom using the “car” package (Fox and Monette, 2002).

When the clinical phenotype fixed effect was significant, a *post-hoc* test was conducted to extract the estimate and the significance of each between class difference. This step was processed using “lsmeans” package in R (Lenth, 2016).

### 2.7. Experimental Settings

T1 and DTI images have been used to obtain a structural *N*× *N* connectivity matrix for each MRI. For each feature vector, normalization was applied so that each value was in the real range [0, 1].

The parameter τ was set to 0.35 according to the method described in Kocevar et al. (2016). The model was trained using Adam (Kingma and Ba, 2014) with learning rate 0.001 and early stopping to prevent overfitting. Cross validation with 3-folds was used to provide a more robust evaluation of the model. The quality of the classification was compared by means of the average F-Measure, Precision, and Recall (Powers, 2011) achieved during the cross validation. Wilcoxon-Mann-Whitney test (Wilcoxon, 1945) was conducted to test the differences between the global metrics measured between the patient's groups.

## 3. Results

In the experiments, we trained the GCNN to classify patients given their brain connectivity adjacency matrix representation and the corresponding vector of node descriptors. Furthermore, we trained the GCNN using all the features together (all-graph). Finally, we used a featureless approach, meaning that the no node descriptor is provided.

### 3.1. Local Graph Metrics Analysis

**Figure 1:**
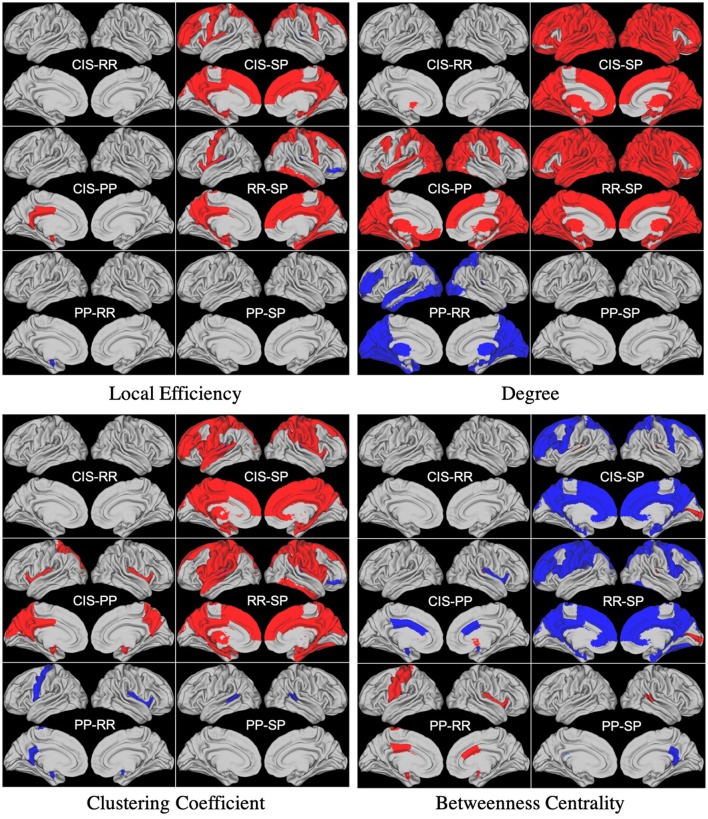
Differences between groups found in statistical analysis performed using unweighted local graph metrics. Blue and Red regions represent negative and positive differences, respectively.

We report results of the statistical analysis performed using unweighted local graph metrics. Many significant differences were found when comparing the betweenness centrality metric of CIS vs. PP and SP as well as when comparing RR vs. PP and SP. No differences were found when comparing CIS vs. RR and PP vs. SP. The same behavior was observed when comparing clustering coefficient, degree, and local efficiency metrics. Moreover, no significant differences were observed when comparing the local efficiency metric of PP vs. RR, except for the left amygdala (*p* < 0.05). Finally, important differences in several regions were found when comparing the local degree of CIS vs. SP and RR vs. SP. An illustration of these results is reported in Figure 1.

**Figure 2:**
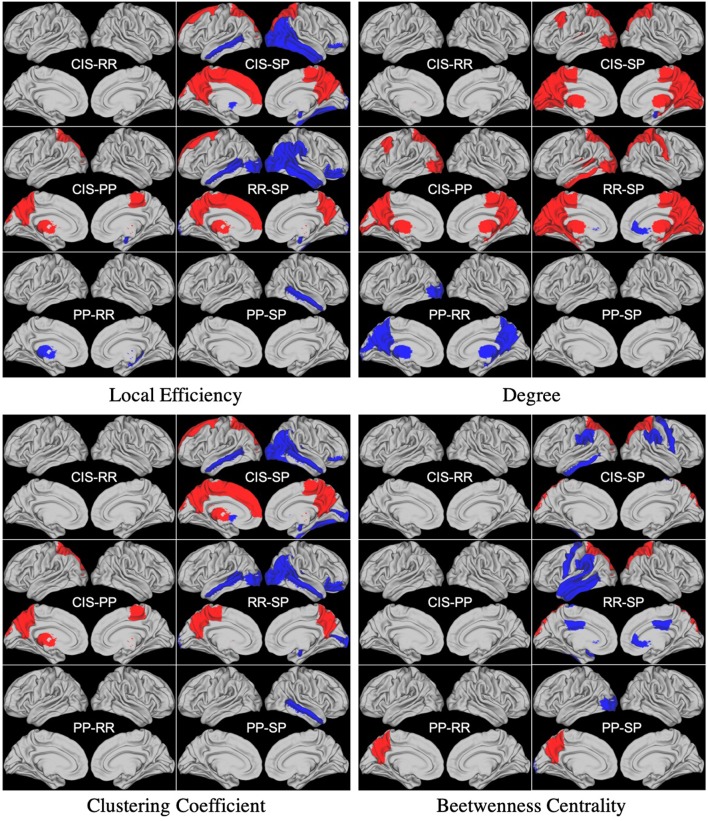
Differences between groups found in statistical analysis performed using weighted local graph metrics. Blue and Red regions represent negative and positive differences, respectively.

Concerning the statistical analysis performed using weighted local graph metrics, several significant differences were found, again, when comparing the betweenness centrality, clustering coefficient, degree, and local efficiency metrics in the CIS and RR groups with respect to the PP and SP groups. No significant differences were observed between CIS and RR except for the degree of the left-caudate nucleus (*p* < 0.05). Concerning the comparison between PP and SP groups, significant differences were found when comparing the betweenness centrality in the left lateral-occipital region and the left precuneus (parietal lobe) and clustering coefficient and efficiency in the right middle-temporal region (*p* < 0.05). This latter region was also found to be the only one differing between PP and SP groups in terms of clustering coefficient (*p* < 0.05). Statistical analysis results performed using weighted local graph metrics are reported in Figure 2.

Detailed results about our statistical analysis are reported as Supplementary Material to this paper.

### 3.2. Classification Using Unweighted Adjacency Matrix

**Figure 3:**
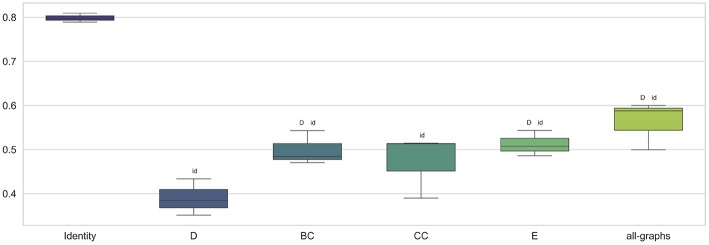
Box plot in term of F-Measure for each different unweighted feature [Degree (D), Betweenness Centrality (BC), Clustering Coefficient (CC), Local Efficiency (E), with all graph-metrics (all-graphs)] and without features (identity).

We first trained the proposed GCNN model using unweighted brain connectivity adjacency matrix representations. Results obtained for each experiment are reported in Table 2 and graphically illustrated in Figure 3. As observable, using local graph-derived metrics as node descriptors combined with unweighted graphs, does not provide sufficient information for the classification, achieving an average F-Measure of about 0.50. In particular, the worst performances were obtained for the Degree metric (F-Measure = 0.39±0.03) while slightly better results were observed, on average, with Clustering Coefficient, Betweenness Centrality, and Efficiency. All the graph-metrics together provided, on average, better results. Nevertheless, the most remarkable results were observed with the featureless approach. In this case, we obtained a significant improvement of the performances (F-Measure = 0.80 ± 0.01), stating that the brain structure itself is highly discriminative for the clinical profiles.

**Table 2:** Cross validation results in terms of F-Measure, Precision, and Recall (± standard deviation) averaged on 3-folds.

	**Identity**	**D**	**BC**	**CC**	**E**	**All-graphs**
**UNWEIGHTED**						
F-Measure	0.80 (±0.01)	0.39 (±0.03)	0.50 (±0.03)	0.47 (±0.06)	0.51 (±0.02)	0.56 (±0.04)
Precision	0.81 (±0.01)	0.33 (±0.03)	0.54 (±0.08)	0.47 (±0.11)	0.56 (±0.06)	0.57 (±0.04)
Recall	0.80 (±0.01)	0.48 (±0.04)	0.55 (±0.04)	0.55 (±0.04)	0.56 (±0.03)	0.60 (±0.04)
**WEIGHTED**						
F-Measure	0.92 (±0.02)	0.64 (±0.01)	0.68 (±0.01)	0.64 (±0.02)	0.62 (±0.03)	0.74 (±0.02)
Precision	0.93 (±0.02)	0.70 (±0.02)	0.69 (±0.01)	0.66 (±0.01)	0.64 (±0.05)	0.76 (±0.02)
Recall	0.93 (±0.02)	0.65 (±0.02)	0.69 (±0.01)	0.64 (±0.02)	0.63 (±0.03)	0.75 (±0.03)

*Rows report achieved results using unweighted graphs with unweighted features (upper) and using weighted graphs with weighted features (lower) [Degree (D), Betweenness Centrality (BC), Clustering Coefficient (CC), Local Efficiency (E), with all graph-metrics (all-graphs)] and without features (identity)*.

### 3.3. Classification Using Weighted Adjacency Matrix

**Figure 4:**
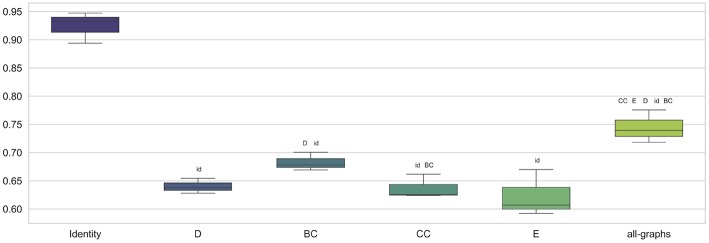
Box plot in term of F-Measure for each different weighted feature [Degree (D), Betweenness Centrality (BC), Clustering Coefficient (CC), Local Efficiency (E), with all graph-metrics (all-graphs)] and without features (identity).

**Figure 5:**
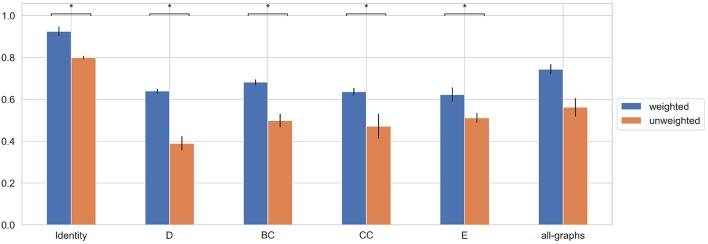
Average F-Measure comparison for weighted and unweighted approach for each feature [Degree (D), Betweenness Centrality (BC), Clustering Coefficient (CC), Local Efficiency (E), with all graph-metrics (all-graphs)] and without features (identity). ^*^Represents statistical significance between the two groups.

We trained the proposed model using weighted brain connectivity adjacency matrix representations. Results obtained for each experiment are provided in Table 2 and reported graphically in Figure 4. Interestingly, good results were achieved (F-Measure > 0.60) with all the studied local graph-metrics. Furthermore, a significant increase in performances was observed when using all the studied features together. Again, the best result was achieved using the featureless approach (F-Measure = 0.92 ± 0.02), with an average F-Measure increasing of 10% with respect to the unweighted version. In particular, a general performance increase can be observed when using weights information, as graphically shown in Figure 5. Indeed, for all the proposed experiments, except when we use all the graph metrics together, a significant improvement is achieved.

### 3.4. Classification of Control Subjects vs. MS Patients

In this section we explore the capability of the proposed models to discriminate between HC and MS patients. More in detail, we performed three main experiments. First, we trained the proposed GCNN model at classifying patients at early stages of the pathology (CIS and RR) from HC. Then, we trained the model at classifying patients at progressive stages (SP vs. PP) from HC. Finally, we performed a multiclass classification task including all the clinical forms, i.e., HC vs. SP vs. PP vs. RR vs. CIS. As for the other experiments in this study, we compared performances using weighted and unweighted brain connectivity adjacency matrix representations.

#### 3.4.1. HC vs. (CIS + RR)

**Figure 6:**
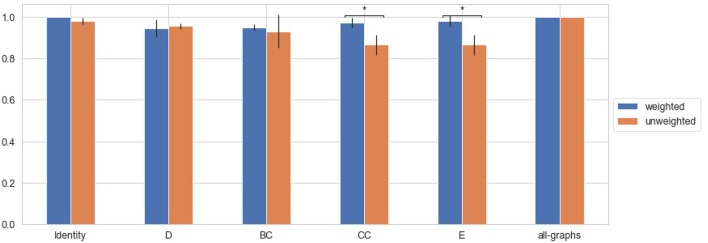
Average F-Measure comparison for weighted and unweighted approach [HC vs. (CIS+RR)] for each feature [Degree (D), Betweenness Centrality (BC), Clustering Coefficient (CC), Local Efficiency (E), with all graph-metrics (all-graphs)] and without features (identity). ^*^Represents statistical significance between the two groups.

HC vs. (CIS + RR) classification task is described in this section. Results obtained using unweighted and weighted connectivity representations are reported in Table 3. Results are then compared graphically in Figure 6. As observable, both weighted and unweighted local graph-derived metrics provide sufficient information for distinguish between HC and MS patients. However, weighted connectivity matrices provide overall better results. Best performances were achieved using no node descriptions and using all graph-metrics together.

**Table 3:** Cross validation results of HC vs. (CIS+RR) in terms of F-Measure, Precision, and Recall (± standard deviation) averaged on 3-folds.

	**Identity**	**D**	**BC**	**CC**	**E**	**All-graphs**
**UNWEIGHTED**						
F-Measure	0.96 (±0.05)	0.93 (±0.06)	0.87 (±0.14)	0.89 (±0.11)	0.89 (±0.11)	1.0 (±0.0)
Precision	1.0 (±0.01)	0.97 (±0.03)	0.99 (±0.01)	0.99 (±0.01)	0.99 (±0.01)	1.0 (±0.0)
Recall	0.94 (±0.08)	0.91 (±0.09)	0.83 (±0.16)	0.85 (±0.14)	0.85 (±0.14)	1.0 (±0.0)
**WEIGHTED**						
F-Measure	1.0 (±0.0)	0.96 (±0.01)	0.96 (±0.04)	0.98 (±0.01)	0.98 (±0.01)	1.0 (±0.0)
Precision	1.0 (±0.0)	0.98 (±0.02)	0.97 (±0.03)	1.0 (±0.0)	1.0 (±0.0)	1.0 (±0.0)
Recall	1.0 (±0.0)	0.95 (±0.03)	0.94 (±0.05)	0.96 (±0.03)	0.96 (±0.03)	1.0 (±0.0)

*Rows report achieved results using unweighted graphs with unweighted features (upper) and using weighted graphs with weighted features (lower) [Degree (D), Betweenness Centrality (BC), Clustering Coefficient (CC), Local Efficiency (E), with all graph-metrics (all-graphs)] and without features (identity)*.

#### 3.4.2. HC vs. (SP + PP)

**Figure 7:**
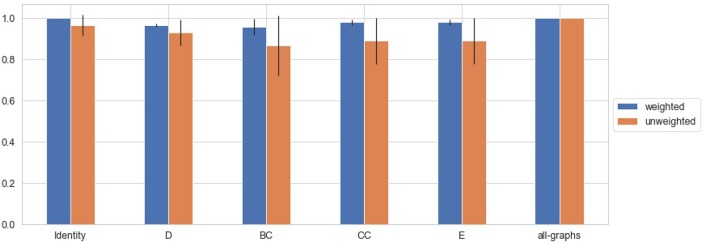
Average F-Measure comparison for weighted and unweighted approach [HC vs. (SP+PP)] for each feature [Degree (D), Betweenness Centrality (BC), Clustering Coefficient (CC), Local Efficiency (E), with all graph-metrics (all-graphs)] and without features (identity).

HC vs. (SP + PP) classification task is described in this section, in order to test the capability of the model in discriminating HC from progressive MS. Results obtained using unweighted and weighted connectivity representations are reported in Table 4. Results are then compared graphically in Figure 7. Again, high level of accuracy were obtained using weighted and unweighted information and weighted connectivity matrices provide overall better results. As for the HC vs. (CIS + RR) task, the highest performances were achieved using no node descriptions and using all graph-metrics together.

**Table 4:** Cross validation results of HC vs. (SP+PP) in terms of F-Measure, Precision, and Recall (± standard deviation) averaged on 3-folds.

	**Identity**	**D**	**BC**	**CC**	**E**	**All-graphs**
**UNWEIGHTED**						
F-Measure	0.96 (±0.05)	0.93 (±0.06)	0.87 (±0.14)	0.89 (±0.11)	0.89 (±0.11)	1.0 (±0.0)
Precision	1.0 (±0.01)	0.97 (±0.03)	0.99 (±0.01)	0.99 (±0.01)	0.99 (±0.01)	1.0 (±0.0)
Recall	0.94 (±0.08)	0.91 (±0.09)	0.83 (±0.16)	0.85 (±0.14)	0.85 (±0.14)	1.0 (±0.0)
**WEIGHTED**						
F-Measure	1.0 (±0.0)	0.96 (±0.01)	0.96 (±0.04)	0.98 (±0.01)	0.98 (±0.01)	1.0 (±0.0)
Precision	1.0 (±0.0)	0.98 (±0.02)	0.97 (±0.03)	1.0 (±0.0)	1.0 (±0.0)	1.0 (±0.0)
Recall	1.0 (±0.0)	0.95 (±0.03)	0.94 (±0.05)	0.96 (±0.03)	0.96 (±0.03)	1.0 (±0.0)

*Rows report achieved results using unweighted graphs with unweighted features (upper) and using weighted graphs with weighted features (lower) [Degree (D), Betweenness Centrality (BC), Clustering Coefficient (CC), Local Efficiency (E), with all graph-metrics (all-graphs)] and without features (identity)*.

#### 3.4.3. All Classes Classification

**Figure 8:**
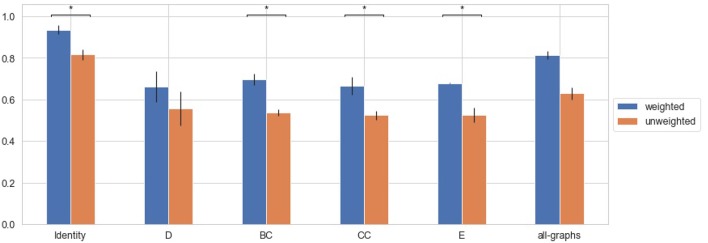
Average F-Measure comparison for weighted and unweighted approach [HC vs. SP vs. PP vs. RR vs. CIS] for each feature [Degree (D), Betweenness Centrality (BC), Clustering Coefficient (CC), Local Efficiency (E), with all graph-metrics (all-graphs)] and without features (identity). ^*^Represents statistical significance between the two groups.

Finally a multiclass classification task is performed using all the forms together. Results obtained using unweighted and weighted connectivity representations are reported in Table 5. Results are then compared graphically in Figure 8. All the graph-metrics together provided, on average, better results. Higher performances were observed performing a featureless approach.

**Table 5:** Cross validation results of HC vs. SP vs. PP vs. RR vs. CIS in terms of F-Measure, Precision, and Recall (± standard deviation) averaged on 3-folds.

	**Identity**	**D**	**BC**	**CC**	**E**	**All-graphs**
**UNWEIGHTED**						
F-Measure	0.82 (±0.03)	0.56 (±0.08)	0.54 (±0.01)	0.52 (±0.02)	0.53 (±0.03)	0.63 (±0.03)
Precision	0.83 (±0.02)	0.72 (±0.04)	0.58 (±0.03)	0.61 (±0.1)	0.59 (±0.03)	0.71 (±0.01)
Recall	0.81 (±0.03)	0.56 (±0.07)	0.53 (±0.01)	0.54 (±0.03)	0.52 (±0.04)	0.63 (±0.02)
**WEIGHTED**						
F-Measure	0.94 (±0.02)	0.66 (±0.07)	0.7 (±0.03)	0.66 (±0.04)	0.68 (±0.0)	0.81 (±0.02)
Precision	0.94 (±0.02)	0.74 (±0.02)	0.75 (±0.03)	0.68 (±0.04)	0.7 (±0.02)	0.84 (±0.01)
Recall	0.93 (±0.02)	0.66 (±0.06)	0.68 (±0.02)	0.67 (±0.04)	0.68 (±0.02)	0.8 (±0.03)

*Rows report achieved results using unweighted graphs with unweighted features (upper) and using weighted graphs with weighted features (lower) [Degree (D), Betweenness Centrality (BC), Clustering Coefficient (CC), Local Efficiency (E), with all graph-metrics (all-graphs)] and without features (identity)*.

### 3.5. Early vs. Progressive Forms Comparison

In this section we explore the capability of the proposed models to discriminate between CIS and RR and progressive MS clinical forms. The proposed GCNN model was trained at distinguishing between CIS-RR and SP-PP patients, in order to provide a better understanding of the different pathophysiology of patients at an early stage of the disease (CIS and RR) and patients with progressive MS. Furthermore, performances using weighted and unweighted brain connectivity adjacency matrix representations were compared.

**Figure 9:**
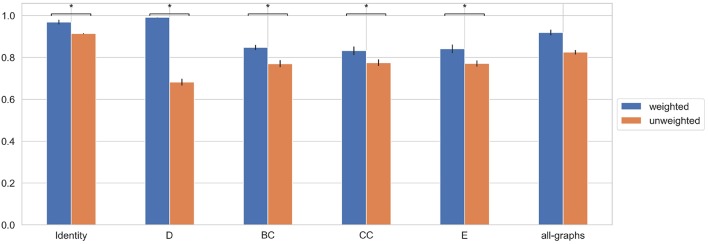
Average F-Measure comparison for weighted and unweighted approach [(CIS+RR) vs. (SP+PP)] for each feature [Degree (D), Betweenness Centrality (BC), Clustering Coefficient (CC), Local Efficiency (E), with all graph-metrics (all-graphs)] and without features (identity). ^*^Represents statistical significance between the two groups.

Results obtained using unweighted and weighted connectivity representations are reported in Table 6. Results are then compared graphically in Figure 9.

**Table 6:** Cross validation results of (CIS+RR) vs. (SP+PP) in terms of F-Measure, Precision, and Recall (± standard deviation) averaged on 3-folds.

	**Identity**	**D**	**BC**	**CC**	**E**	**All-graphs**
**UNWEIGHTED**						
F-Measure	0.91 (±0.1)	0.68 (±0.02)	0.77 (±0.02)	0.78 (±0.02)	0.77 (±0.01)	0.83 (±0.01)
Precision	0.92 (±0.1)	0.7 (±0.01)	0.78 (±0.01)	0.78 (±0.01)	0.78 (±0.02)	0.83 (±0.01)
Recall	0.91 (±0.0)	0.68 (±0.02)	0.77 (±0.02)	0.78 (±0.02)	0.77 (±0.02)	0.83 (±0.01)
**WEIGHTED**						
F-Measure	0.97 (±0.01)	0.99 (±0.0)	0.85 (±0.01)	0.83 (±0.02)	0.84 (±0.02)	0.92 (±0.01)
Precision	0.97 (±0.01)	0.99 (±0.0)	0.85 (±0.01)	0.84 (±0.02)	0.85 (±0.02)	0.92 (±0.01)
Recall	0.97 (±0.01)	0.99 (±0.0)	0.85 (±0.01)	0.83 (±0.02)	0.84 (±0.02)	0.92 (±0.01)

*Rows report achieved results using unweighted graphs with unweighted features (upper) and using weighted graphs with weighted features (lower) [Degree (D), Betweenness Centrality (BC), Clustering Coefficient (CC), Local Efficiency (E), with all graph-metrics (all-graphs)] and without features (identity)*.

An overall increase in performances were observed respect to the intra clinical form classification tasks previously performed. Both weighted and unweighted local graph-derived metrics provide promising results. Interestingly, weighted Degree allow to achieve high level of accuracy; indeed, it is worth to note that graph density decreases along with the progress of the pathology, due to neurodegenerative processes. However, as for previous experiments, the featureless approach provided better and more stable results.

## 4. Discussions

In this work, we proposed a novel graph-based neural network method to classify MS patients according to their clinical phenotype using brain structural connectivity information. To this aim, we exploited a peculiar type of neural network architecture designed to handle arbitrarily structured graphs. We compared the impact of local graph metrics to the classification performances, either using weighted and unweighted brain connectivity representation. Furthermore, we performed a statistical analysis on the local graph metrics (weighted and unweighted) computed for each MS clinical form, attempting to characterize differences in the groups, and eventually classify patients. Notice that, conventional MRI information such as lesion and gray matter volumes did not allowed to accurately classify patients into the four clinical forms, thus showing the lack of specificity of these measurements to the pathophysiological effects of the disease. Details about the analysis can be found in the Supplementary Material.

Our attention focalized on the classification of the MS clinical courses, differently from previous related works which mostly focused on the differentiation between MS patients from HC subjects (Maleki et al., 2012; Zhang et al., 2018; Zurita et al., 2018; Charalambous et al., 2019). The task addressed in this work has a strong clinical interest as the early clinical classification and thus prognostic of MS patients is the major challenge for neurologists today. Indeed, it is worth recalling that MS etiology is still unknown and that each MS patient may follow a different clinical course, resulting probably from the variety of the underground pathological mechanisms. More in detail, CIS and RR patients present comparable brain pathological processes, mainly inflammation, while SP and PP patients share neurodegenerative mechanisms. Indeed, as showed by the statistical analysis, few differences were found when comparing local graph metrics between these two pair of groups. As for the CIS vs. RR comparison, these differences are mostly localized in the sub-cortical regions, temporal and parietal lobes, highlighting that early pathological processes start in central subcortical structures. Also, these differences are more related to weighted measures, thus showing that inflammation has a stronger impact on large WM fiber bundles.

Concerning the SP vs. PP comparison, very few differences were found in the occipital, parietal, and temporal lobes, reflecting the similarity effect of the neurodegenerative process. Significant differences were mainly observed when comparing early stages of the disease (CIS,RR) with more severe clinical forms (SP,PP). As previously observed in literature (Kocevar et al., 2016; Charalambous et al., 2019), indeed, a general reduction in network efficiency, density and clustering coefficient was observed in SP relative to RR patients due to severe brain damages. Finally, is interesting to notice the similarity between differences found in local efficiency and clustering coefficient (weighted). This result is in agreement with a recent study (Strang et al., 2018), where these two metrics were found to be asymptotically linearly correlated in functional connectivity and various benchmark graphs.

However, despite difficulties in discriminating among groups using statistical markers, the proposed approaches achieved promising results. The proposed GCNN architecture was able to achieve good results operating with a relatively small number of parameters (about 42,000 trainable weights) compared with classical convolutional networks models working on images. Indeed, it is worth to note that the proposed method works on adjacency matrices of 84 nodes, significantly reducing the number of input units with respect to models directly operating on MR images (Maleki et al., 2012; Zhang et al., 2018).

Interestingly, the main results were obtained using only the connectivity matrices, without graph-metrics as node descriptor. The brain structure, indeed, seems to contain highly discriminative properties characterizing the clinical profiles. Some of these properties are already mentioned in literature. According to the statistical analysis performed in Kocevar et al. (2016), for example, significant differences were found when comparing several graph metrics in progressive courses, reflecting the neurodegenerative mechanisms acting in the brain. However, as shown in our experiments, none of these properties used as node descriptors were effectively exploited by the neural network to discriminate the groups. By contrast, interesting improvement in classification performances were observed when using all the graph metrics together. The result is in agreement with a previous study (Kocevar et al., 2016) where the combination of global graph-derived metrics provided the best results in the CIS vs. RR, RR vs. PP, CIS vs. RR vs. SP classification tasks. This may be explained considering that each local descriptor can provide useful information for a particular clinical course. Thus, exploiting them all together allows a better characterization of MS pathological alterations.

Interesting results were also observed when evaluating the capability of the network in classifying “early" stages of the pathology vs. “progressive" stages. The proposed model was able to perform the binary classification task achieving high level of accuracy. Remarkable results were obtained considering the weighted Degree, which allowed the model to achieve the best performance. Indeed, it is worth to note that graph density decreases along with the progress of the pathology, due to neurodegenerative processes (Kocevar et al., 2016; Charalambous et al., 2019).

Experiments including control patients were also reported in this paper. The proposed architecture was trained at discriminating between HC and early stages of the pathology (CIS and RR), and between HC and progressive stages (PP and SP). The model was able to achieve high performances. HC subjects were also used to perform a multiclass classification task using all the forms together. All the graph-metrics together as well as the featureless approach provided, on average, better results, confirming our previous observations. These results are somehow straightforward. Related studies have already shown several differences comparing brain structure representation of control subjects with respect to MS patients (Zurita et al., 2018; Charalambous et al., 2019), due to pathological alterations. Such effects, cause HC networks to be more dense and well organized compared to MS, thus allowing an accurate discrimination (Kocevar et al., 2016). However, even if expected, these further analyses confirm the capability of the model to detect and exploit brain structure differences.

Finally, one of the main observation is related to the significant role played by edge weights in the classification task. As shown in our results, weights information allowed significant performance improvements in almost all the experiments. This achievement suggests that, despite comparable alterations in white matter network structure among groups may lead to misclassification in some cases, the fiber bundles' strength provides a complementary information helpful to improve the overall accuracy.

## 5. Conclusions

In this work, we proposed a graph-based method to classify MS patients according to their clinical forms. Graph theory has been applied to describe brain network topology and Graph Convolutional Neural Networks have been used for the classification of MS clinical courses.

Thanks to a robust experimental activity, we showed the capability of GCNN to classify MS patients using the whole graph structure. In order to have a clear picture, we also enriched our analysis by combining the graph structure information with local graph-based metrics. Three major results were achieved by this analysis:(i) NNs are able to achieve high classification results using only the connectivity matrix (ii) local graph metrics do not improve the classification results suggesting that the latent features created by the NN in its layers have a much important informative content (iii) graph weights representation of brain connections preserve important information to discriminate between clinical forms. This result suggests that with graph binarization a lot of useful information may be lost.

It is worth to note that a limitation of this study is the small number of patients. However, we minimized these potential biases by using K-Fold cross-validation to generalize classification results. Further, the small number of each patient profile may not reflect the general population and induce biases in graph metrics results. As for future work, we aim at improving our method using a whole trail of longitudinal data collected for each patient as input for the model. In order to carry out this task, novel models proposed in literature may be taken into account. In this context, Recurrent Neural Networks (Medsker and Jain, 1999) have achieved remarkable results in dealing with short-long temporal relations (Graves and Jaitly, 2014; Donahue et al., 2015; Fragkiadaki et al., 2015) and can be effectively extended to handle graph data (Jain et al., 2016; Manessi et al., 2017; Jin and JaJa, 2018), achieving promising results. Another interesting perspective would be to perform a deep clinical analysis in order to understand the potential interest of such methods to better characterize the disease progression and thus better predict the patient evolution.

## Data Availability

The datasets for this manuscript are not publicly available because The dataset used in this study belong to the OFSEP consortium. Requests to access the datasets should be directed to Dominique Sappey-Marinier, dominique.sappey-marinier@univ-lyon1.fr.

## Ethics Statement

This study was carried out in accordance with the recommendations of the French national agency for medicine and health products safety (ANSM) with written informed consent from all subjects. All subjects gave written informed consent in accordance with the Declaration of Helsinki. The protocol was approved by the local ethics committee (CPP Sud-Est IV).

## Author Contributions

AM developed the method, performed the classification, and wrote the paper. GK performed the statistical analysis and wrote the paper. FD-D provided the data and helped in the interpretation of the results. FC, GT, CS, and DS-M wrote the paper, provided the technical, and clinical expertise in the interpretation of the results and designed the study.

### Conflict of Interest Statement

The authors declare that the research was conducted in the absence of any commercial or financial relationships that could be construed as a potential conflict of interest.

## Footnotes

^1^Brain Connectivity Toolbox: https://sites.google.com/site/bctnet/
