# Supplementary material for: Classification of Multiple Sclerosis Clinical Profiles via Graph Convolutional Neural Networks
Source: Front Neurosci. 2019 Jun 12;13:594. doi: 10.3389/fnins.2019.00594 (PMC6581753; doi:10.3389/fnins.2019.00594)
Supplement: Supplementary file 1 [file Data_Sheet_1.PDF]

Supplementary Material

1 CONVENTIONAL MRI DATA ANALYSIS

In this section we analyze conventional MRI information (T2 lesions and grey matter volumes) in order to investigate whether such information are sufficient to discriminate MS clinical forms. Gray matter and lesions were segmented based on T2 FLAIR, using the MSmetrix software developed by icometrix (Leuven, Belgium).

1.1 Statistical Analysis

A linear mixed-effects model was applied separately to lesions and grey matter volumes:

$$Response_{ij} = \beta_0 + \beta_1(Clinical\ phenotype_i) + \beta_2(Scan\ Session_{ij}) + b_{0i} + \epsilon_{ij} \tag{S1}$$

In this model, the predicted response of interest for subject i at time j is determined by fixed effects, represented by β_1 and β_2. Subject-specific effects are represented by b_{0i}, allowing a random interception per subject i. When the clinical phenotype fixed effect was significant, a post-hoc test was conducted to extract the estimate and the significance of each between class difference. Results are illustrated in Table S1 and Table S2. Significant differences were found comparing grey matter volumes of CIS with PP $(p < 0.01)$ and SP $(p < 0.01)$ and comparing grey matter volumes of RR with SP $(p < 0.01)$ and PP $(p < 0.05)$. Furthermore, significant differences were found comparing lesions volumes of CIS with PP $(p < 0.05)$ and SP $(p < 0.001)$ and comparing lesions volumes of SP with RR $(p < 0.001)$ and PP $(p < 0.05)$.

Table S1. Statistical analysis results using T2 lesions volumes

contrast	estimate	t-ratio	p-value
CIS - PP	-13.85	-3.10	0.0139 *
CIS - RR	-10.73	-2.60	0.0524
CIS - SP	-24.11	-5.76	<0.0001 ***
PP - RR	3.12	0.88	0.8132
PP - SP	-10.26	-2.85	0.0278 *
RR - SP	-13.40	-4.24	0.0003 ***

* p<0.05; ** p<0.01; *** p<0.001

Table S2. Statistical analysis results using grey matter volumes

contrast	estimate	t-ratio	p-value
CIS - PP	49.24	3.32	0.0072 **
CIS - RR	17.24	1.26	0.5910
CIS - SP	53.44	3.85	0.0013 **
PP - RR	-32.01	-2.73	0.0376 *
PP - SP	4.30	0.35	0.9850
RR - SP	36.21	3.46	0.0046 **

* p<0.05; ** p<0.01; *** p<0.001

1.2 Classification using Naïve Approach

Despite statistical differences among MS clinical forms in terms of lesions volumes and grey matter volumes, such information might not be sufficient to discriminate patients for developing an automated classification method. A naïve classifier was defined in order to investigate the discrimination capability of conventional MRI information. A randomly selected seventy percent of the dataset was used to compute the mean of each group. Then, to each sample of the test set is assigned the class corresponding to the closer mean. The experiments was repeated $k = 100$ times and results using lesions volumes and grey matter are reported in Table S3 and Table S4, respectively.

Table S3. Naïve classification results for the multiclass classification task using T2 lesions volumes

	mean (± std)
F-Measure	0.37 ± 0.02
Accuracy	0.42 ± 0.03
Precision	0.37 ± 0.03
Recall	0.48 ± 0.02

Table S4. Naïve classification results for the multiclass classification task using grey matter volumes

	mean (± std)
F-Measure	0.31 ± 0.03
Accuracy	0.32 ± 0.03
Precision	0.32 ± 0.03
Recall	0.38 ± 0.03

1.3 Classification using Support Vector Machine

A Support Vector Machine (SVM) was trained to classify among the MS clinical forms using lesions and grey matter volumes. In order to find the optimal input parameters of SVM, namely C and γ, grid search was performed using growing sequences of C and γ. More in detail, we used the range [0.001,10] for C and [0.001, 1] for γ. Both linear and radial basis function (rbf) kernel were tested. Generalization of classification performances was ensured by K-Fold cross validation using 3-folds. Finally, each feature was standardized in order to improve the quality of the classification. Perfomances was evaluated by means of F-Measure, Accuracy, Precision and Recall. Best results were obtained using linear kernel with $C = 0.01$, and $\gamma = 0.001$ and reported in Table S5. As observable, conventional MRI data do not provide sufficient information to correctly perform the multiclass classification task. Indeed, despite statistical differences among groups, the number of overlapping values is too high to discriminate among clinical statuses.

Table S5. SVM classification results for the multiclass classification task (CIS vs RR vs SP vs PP) using conventional MRI information

	mean (± std)
F-Measure	0.27 ± 0.03
Accuracy	0.47 ± 0.04
Precision	0.24 ± 0.02
Recall	0.34 ± 0.03

2 SUPPLEMENTARY DATA

Detailed results for the statistical analysis are reported in this section. The following tables show differences between each pair of MS clinical forms Values represent differences between each pair of MS clinical forms (CIS vs RR, CIS vs SP, CIS vs PP, RR vs SP, PP vs RR, PP vs SP).

Table S6. Detailed results for the unweighted betweenness centrality. Values represent differences between each pair of MS clinical forms (CIS vs RR, CIS vs SP, CIS vs PP, RR vs SP, PP vs RR, PP vs SP). Statistical significance is also reported.

			CIS vs RR	CIS vs SP	CIS vs PP	RR vs SP	PP vs RR	PP vs SP
2	Left caudalanteriorcingulate	Cingulate		-10.74 *	-10.73 *			
5	Left entorhinal	Temporal		-4.48 *		-3.63 *		
9	Left isthmuscingulate	Cingulate		-26.78 **		-19.33 **		
15	Left parahippocampal	Temporal						
17	Left parsopercularis	Frontal		-9.95 *		-8.06 *		
21	Left postcentral	Parietal				-6.34 *	6.19 *	
22	Left posteriorcingulate	Cingulate		-28.34 **	-22.97 *	-21.14 ***	15.77 *	
23	Left precentral	Frontal		-18.81 **		-17.36 ***	12.86 *	
24	Left precuneus	Parietal		-53.52 **		-39.74 **		
25	Left rostralanteriorcingulate	Cingulate		-5.62 *		-4.80 **		
26	Left rostralmiddlefrontal	Frontal		-26.10 **		-16.61 *		
27	Left superiorfrontal	Frontal		-47.32 *		-33.79 *		
28	Left superiorparietal	Parietal		-43.69 **		-29.02 *		
30	Left supramarginal	Parietal				-7.08 *		
33	Left transversetemporal	Temporal		2.42 *				
34	Left insula	Insula						
35	Left-Cerebellum-Cortex	Cerebellum				-56.32 *		
38	Left-Putamen	Sub-cortical GM		-29.78 **		-24.22 **		
39	Left-Pallidum	Sub-cortical GM				-18.20 *		
40	Left-Hippocampus	Sub-cortical GM		-50.73 *		-42.40 **		
41	Left-Amygdala	Sub-cortical GM			-8.08 *		8.60 **	
44	Right-Caudate	Sub-cortical GM				-9.29 *		
45	Right-Putamen	Sub-cortical GM				-23.49 **		
46	Right-Pallidum	Sub-cortical GM		-25.08 *		-26.70 ***		
47	Right-Hippocampus	Sub-cortical GM		-52.86 **		-43.28 **		
48	Right-Amygdala	Sub-cortical GM		-11.29 *				
49	Right-Accumbens-area	Sub-cortical GM			3.82 *			
50	Right bankssts	Temporal						4.32 *
51	Right caudalanteriorcingulate	Cingulate		-18.48 **	-15.75 *	-15.78 **	13.05 *	
54	Right entorhinal	Temporal		-6.36 *		-6.54 **		
55	Right fusiform	Temporal				-6.79 **		
58	Right isthmuscingulate	Cingulate		-29.48 **		-23.45 **		-20.56 *
66	Right parsopercularis	Frontal				-6.28 *		
69	Right pericalcarine	Occipital		6.30 **		4.07 **		
71	Right posteriorcingulate	Cingulate		-28.81 ***		-23.25 ***		
72	Right precentral	Frontal		-19.15 **		-18.20 ***		
73	Right precuneus	Parietal		-48.21 **		-29.55 *		
74	Right rostralanteriorcingulate	Cingulate		-7.77 *		-6.91 **		
76	Right superiorfrontal	Frontal		-40.55 **		-34.15 ***		
77	Right superiorparietal	Parietal		-47.66 **		-33.40 **		
82	Right transversetemporal	Temporal		2.50 ***	1.80 *	1.53 **		
83	Right insula	Insula			-32.27 *		32.91 **	
84	Right-Cerebellum-Cortex	Cerebellum						

* $p<0.05$; ** $p<0.01$; *** $p<0.001$

Table S7. Detailed results for the unweighted clustering coefficient. Values represent differences between each pair of MS clinical forms (CIS vs RR, CIS vs SP, CIS vs PP, RR vs SP, PP vs RR, PP vs SP). Statistical significance is also reported.

			CIS vs RR	CIS vs SP	CIS vs PP	RR vs SP	PP vs RR	PP vs SP
	Left bankssts	Temporal						-0.03 *
2	Left caudalanteriorcingulate	Cingulate						
4	Left cuneus	Occipital			0.05 *			
5	Left entorhinal	Temporal		0.07 *		0.07 **		
7	Left inferiorparietal	Parietal						
9	Left isthmuscingulate	Cingulate		0.09 ***	0.07 **	0.07 ***	-0.05 *	
15	Left parahippocampal	Temporal				0.05 *		
16	Left paracentral	Frontal		0.06 *				
17	Left parsopercularis	Frontal		0.04 *		0.03 *		
21	Left postcentral	Parietal		0.04 *		0.04 **		
22	Left posteriorcingulate	Cingulate		0.09 ***	0.07 **	0.06 ***		
23	Left precentral	Frontal		0.06 **		0.05 ***	-0.04 *	
24	Left precuneus	Parietal		0.08 **	0.06 *	0.06 **		
25	Left rostralanteriorcingulate	Cingulate						
26	Left rostralmiddlefrontal	Frontal		0.05 **				
27	Left superiorfrontal	Frontal		0.06 **		0.04 *		
28	Left superiorparietal	Parietal		0.07 **	0.05 *	0.04 *		
29	Left superiortemporal	Temporal		0.04 *		0.03 *		
30	Left supramarginal	Parietal				0.03 *		
33	Left transversetemporal	Temporal		-0.04 *		-0.03 *		
34	Left insula	Insula		0.05 **	0.04 *	0.04 **		
35	Left-Cerebellum-Cortex	Cerebellum		0.08 **		0.06 *		
36	Left-Thalamus-Proper	Sub-cortical GM		0.04 *		0.03 *		
38	Left-Putamen	Sub-cortical GM		0.05 **	0.04 *	0.04 **		
39	Left-Pallidum	Sub-cortical GM						
40	Left-Hippocampus	Sub-cortical GM		0.08 **		0.06 **		
41	Left-Amygdala	Sub-cortical GM		0.05 *	0.06 **	0.04 **	-0.05 **	
43	Right-Thalamus-Proper	Sub-cortical GM						
44	Right-Caudate	Sub-cortical GM				0.03 **		
45	Right-Putamen	Sub-cortical GM				0.04 **		
46	Right-Pallidum	Sub-cortical GM		0.05 *		0.05 **		
47	Right-Hippocampus	Sub-cortical GM		0.09 **		0.06 **		
48	Right-Amygdala	Sub-cortical GM		0.06 **	0.06 *	0.04 *		
50	Right bankssts	Temporal						-0.03 **
51	Right caudalanteriorcingulate	Cingulate		0.06 **		0.04 **		
53	Right cuneus	Occipital				0.04 *		
54	Right entorhinal	Temporal		0.08 *		0.08 **		
55	Right fusiform	Temporal				0.05 **		
56	Right inferiorparietal	Parietal						
57	Right inferiortemporal	Temporal				0.03 *		
58	Right isthmuscingulate	Cingulate		0.10 ***		0.07 ***		
63	Right middletemporal	Temporal						
64	Right parahippocampal	Temporal		0.09 **		0.07 **		
65	Right paracentral	Frontal		0.05 *				
66	Right parsopercularis	Frontal				0.03 *		
67	Right parsorbitalis	Frontal				-0.04 **		
70	Right postcentral	Parietal		0.04 *		0.03 *		
71	Right posteriorcingulate	Cingulate		0.09 ***		0.06 **		
72	Right precentral	Frontal		0.06 **		0.04 **		
73	Right precuneus	Parietal		0.08 ***	0.06 *	0.06 **		
74	Right rostralanteriorcingulate	Cingulate		0.04 *				
76	Right superiorfrontal	Frontal		0.06 **		0.04 **		
77	Right superiorparietal	Parietal		0.07 **		0.05 **		
79	Right supramarginal	Parietal		0.04 *		0.03 *		
82	Right transversetemporal	Temporal		-0.04 **		-0.03 **		
83	Right insula	Insula		0.04 *	0.04 *	0.03 *	-0.03 *	
84	Right-Cerebellum-Cortex	Cerebellum		0.07 *		0.05 *		

* $p<0.05$; ** $p<0.01$; *** $p<0.001$

Table S8. Detailed results for the unweighted degree. Values represent differences between each pair of MS clinical forms (CIS vs RR, CIS vs SP, CIS vs PP, RR vs SP, PP vs RR, PP vs SP). Statistical significance is also reported.

			CIS vs RR		CIS vs SP		CIS vs PP		RR vs SP		PP vs RR		PP vs SP	
1	Left bankssts	Temporal			7.16	*			7.94	**				
3	Left caudalmiddlefrontal	Frontal			10.75	***	7.21	*	6.37	**				
4	Left cuneus	Occipital			15.27	***	11.20	*	12.92	***	-8.85	*		
6	Left fusiform	Temporal			10.29	**	8.98	*	8.66	***	-7.36	**		
7	Left inferiorparietal	Parietal			13.83	***	10.90	*	10.12	***				
8	Left inferiortemporal	Temporal			10.45	**			10.35	***	-8.46	*		
9	Left isthmuscingulate	Cingulate			11.96	**	8.99	*	10.44	***	-7.47	*		
10	Left lateraloccipital	Occipital			17.25	***	14.96	**	13.77	***	-11.48	**		
11	Left lateralorbitofrontal	Frontal			6.37	*	5.65	*	4.42	*				
12	Left lingual	Occipital			16.44	***	12.12	**	13.75	***	-9.43	**		
13	Left medialorbitofrontal	Frontal			7.93	**	7.03	**						
14	Left middletemporal	Temporal			11.37	*			9.75	**				
15	Left parahippocampal	Temporal			5.84	**			5.64	***	-4.16	*		
16	Left paracentral	Frontal							8.30	***				
19	Left parstriangularis	Frontal			5.90	*								
20	Left pericalcarine	Occipital			13.31	***	10.35	**	10.80	***	-7.83	**		
21	Left postcentral	Parietal			10.53	**	7.82	*	7.53	**				
22	Left posteriorcingulate	Cingulate												
23	Left precentral	Frontal			8.55	**			6.03	*				
24	Left precuneus	Parietal			12.11	**	10.12	*	10.45	***	-8.45	*		
26	Left rostralmiddlefrontal	Frontal			6.16	*			5.56	**	-4.73	*		
27	Left superiorfrontal	Frontal			6.89	**			5.80	**				
28	Left superiorparietal	Parietal			12.68	***	10.22	*	10.98	***	-8.52	*		
29	Left superiortemporal	Temporal			12.95	***	10.07	*	10.12	***	-7.24	*		
30	Left supramarginal	Parietal			9.34	**			5.75	*				
32	Left temporalpole	Temporal			6.51	*	8.69	**	5.45	*	-7.62	**		
33	Left transversetemporal	Temporal			12.33	***	9.23	**	8.54	***	-5.45	*		
34	Left insula	Insula			10.21	**			8.22	**				
35	Left-Cerebellum-Cortex	Cerebellum			9.52	**	7.99	*	8.43	***	-6.90	**		
36	Left-Thalamus-Proper	Sub-cortical GM			12.66	***	10.16	**	9.85	***	-7.35	*		
37	Left-Caudate	Sub-cortical GM			9.92	***	8.94	**	6.88	**	-5.90	*		
38	Left-Putamen	Sub-cortical GM			10.52	**	10.30	*	8.04	**	-7.82	*		
39	Left-Pallidum	Sub-cortical GM			9.33	***	9.22	**	6.89	**	-6.78	**		
40	Left-Hippocampus	Sub-cortical GM			11.67	***	10.45	***	10.13	***	-8.91	***		
41	Left-Amygdala	Sub-cortical GM			5.34	*			3.81	*				
42	Left-Accumbens-area	Sub-cortical GM	5.28	*	7.10	**	8.13	***						
43	Right-Thalamus-Proper	Sub-cortical GM			13.82	***	10.58	**	9.78	***	-6.53	*		
44	Right-Caudate	Sub-cortical GM			8.88	***	7.13	*	4.45	*				
45	Right-Putamen	Sub-cortical GM			12.70	***	10.67	**	7.72	**				
46	Right-Pallidum	Sub-cortical GM			8.94	***	8.40	**	5.78	**	-5.24	*		
47	Right-Hippocampus	Sub-cortical GM			11.79	***	11.13	**	10.51	***	-9.85	***		
48	Right-Amygdala	Sub-cortical GM			4.53	*			4.26	**	-3.63	*		
49	Right-Accumbens-area	Sub-cortical GM			6.03	**	6.93	**						
50	Right bankssts	Temporal			9.96	*			8.68	**				
52	Right caudalmiddlefrontal	Frontal			8.30	**			5.67	*				
53	Right cuneus	Occipital			14.21	***	12.18	**	10.17	***	-8.14	*		
55	Right fusiform	Temporal			11.23	***	9.04	**	7.64	**				
56	Right inferiorparietal	Parietal			16.13	***	11.27	*	11.69	***				
57	Right inferiortemporal	Temporal			10.82	**			9.43	**				
58	Right isthmuscingulate	Cingulate			12.36	***	9.89	*	10.34	***	-7.87	*		
59	Right lateraloccipital	Occipital			18.48	***	13.40	**	14.00	***	-8.93	*		
60	Right lateralorbitofrontal	Frontal			5.97	*			4.21	*				
61	Right lingual	Occipital			16.22	***	11.48	**	12.59	***	-7.86	*		
63	Right middletemporal	Temporal			12.23	**			10.46	**				
64	Right parahippocampal	Temporal			7.17	**			6.92	***	-4.83	*		
65	Right paracentral	Frontal			7.39	*			7.09	**				
67	Right parsorbitalis	Frontal			5.91	**			5.38	***				
69	Right pericalcarine	Occipital			16.65	***	12.52	***	12.22	***	-8.08	**		
70	Right postcentral	Parietal			10.58	**	7.64	*	8.10	***				
72	Right precentral	Frontal			8.81	**	7.27	*	6.47	**				
73	Right precuneus	Parietal			13.98	***	10.09	*	12.29	***	-8.40	*		
75	Right rostralmiddlefrontal	Frontal			8.68	***			6.78	***				
76	Right superiorfrontal	Frontal			7.70	**	6.03	*	5.57	**				
77	Right superiorparietal	Parietal			13.63	***	10.08	*	11.62	***	-8.07	*		
78	Right superiortemporal	Temporal			14.51	***			11.68	***				
79	Right supramarginal	Parietal			9.82	**			7.30	**				
81	Right temporalpole	Temporal					6.70	*	5.22	*	-6.22	*		
82	Right transversetemporal	Temporal			11.69	***	8.69	**	8.51	***	-5.51	*		
83	Right insula	Insula			11.66	**			8.72	**				
84	Right-Cerebellum-Cortex	Cerebellum			9.54	***	7.05	*	8.60	***	-6.11	**		

* p<0.05; * p<0.01; ** p<0.001

Table S9. Detailed results for the unweighted local efficiency. Values represent differences between each pair of MS clinical forms (CIS vs RR, CIS vs SP, CIS vs PP, RR vs SP, PP vs RR, PP vs SP). Statistical significance is also reported.

			CIS vs RR	CIS vs SP	CIS vs PP	RR vs SP	PP vs RR	PP vs SP
5	Left entorhinal	Temporal				0.04 **		
9	Left isthmuscingulate	Cingulate		0.05 ***	0.04 *	0.03 **		
21	Left postcentral	Parietal						
22	Left posteriorcingulate	Cingulate		0.05 ***	0.04 *	0.03 **		
23	Left precentral	Frontal		0.03 **		0.03 **		
24	Left precuneus	Parietal		0.04 **		0.03 **		
26	Left rostralmiddlefrontal	Frontal		0.03 *				
27	Left superiorfrontal	Frontal		0.03 *				
28	Left superiorparietal	Parietal		0.03 **				
33	Left transversetemporal	Temporal				-0.02 *		
34	Left insula	Insula		0.03 *		0.02 *		
35	Left-Cerebellum-Cortex	Cerebellum		0.04 *		0.03 *		
38	Left-Putamen	Sub-cortical GM		0.03 *				
40	Left-Hippocampus	Sub-cortical GM		0.04 **		0.03 **		
41	Left-Amygdala	Sub-cortical GM		0.03 *	0.03 *	0.02 *	-0.02 *	
45	Right-Putamen	Sub-cortical GM				0.02 *		
46	Right-Pallidum	Sub-cortical GM				0.02 *		
47	Right-Hippocampus	Sub-cortical GM		0.05 **		0.03 *		
48	Right-Amygdala	Sub-cortical GM		0.03 *				
51	Right caudalanteriorcingulate	Cingulate		0.03 *		0.02 *		
53	Right cuneus	Occipital						
54	Right entorhinal	Temporal				0.04 *		
55	Right fusiform	Temporal				0.02 *		
57	Right inferiortemporal	Temporal				0.01 *		
58	Right isthmuscingulate	Cingulate		0.05 **		0.04 **		
63	Right middletemporal	Temporal						
64	Right parahippocampal	Temporal		0.05 *		0.04 *		
65	Right paracentral	Frontal						
67	Right parsorbitalis	Frontal				-0.02 **		
71	Right posteriorcingulate	Cingulate		0.04 **		0.03 **		
72	Right precentral	Frontal		0.03 *		0.02 *		
73	Right precuneus	Parietal		0.04 **		0.03 *		
76	Right superiorfrontal	Frontal		0.04 **		0.02 **		
77	Right superiorparietal	Parietal		0.04 **		0.02 *		
82	Right transversetemporal	Temporal		-0.02 *		-0.02 **		
83	Right insula	Insula						
84	Right-Cerebellum-Cortex	Cerebellum		0.04 *				

* $p<0.05$; ** $p<0.01$; *** $p<0.001$

Table S10. Detailed results for the weighted betweenness centrality. Values represent differences between each pair of MS clinical forms (CIS vs RR, CIS vs SP, CIS vs PP, RR vs SP, PP vs RR, PP vs SP). Statistical significance is also reported.

			CIS vs RR	CIS vs SP	CIS vs PP	RR vs SP	PP vs RR	PP vs SP
8	Left inferiortemporal	Temporal		-96.05 **		-63.14 *		
10	Left lateraloccipital	Occipital						-174.11 *
14	Left middletemporal	Temporal				-81.27 **		
22	Left posteriorcingulate	Cingulate				-94.99 **		
23	Left precentral	Frontal				-311.69 *		
24	Left precuneus	Parietal					156.52 *	179.83 *
25	Left rostralanteriorcingulate	Cingulate						
28	Left superiorparietal	Parietal		395.25 *		279.94 *		
29	Left superiortemporal	Temporal				-71.20 **		
30	Left supramarginal	Parietal		-384.99 *		-293.34 *		
71	Right posteriorcingulate	Cingulate				-85.17 **		
72	Right precentral	Frontal		-507.48 **				
74	Right rostralanteriorcingulate	Cingulate				-31.56 *		
77	Right superiorparietal	Parietal		585.17 **		401.12 **		
79	Right supramarginal	Parietal		-412.79 *				

* $p<0.05$; ** $p<0.01$; *** $p<0.001$

Table S11. Detailed results for the weighted clustering coefficient. Values represent differences between each pair of MS clinical forms (CIS vs RR, CIS vs SP, CIS vs PP, RR vs SP, PP vs RR, PP vs SP). Statistical significance is also reported.

			CIS vs RR		CIS vs SP		CIS vs PP		RR vs SP		PP vs RR		PP vs SP	
1	Left bankssts	Temporal							-6.69	*				
6	Left fusiform	Temporal												
8	Left inferiortemporal	Temporal												
10	Left lateraloccipital	Occipital							-7.75	*				
14	Left middletemporal	Temporal			-8.76	*			-7.13	*				
16	Left paracentral	Frontal			6.41	**			4.13	*				
24	Left precuneus	Parietal			8.49	***	6.43	*	5.55	**				
27	Left superiorfrontal	Frontal			8.79	*								
28	Left superiorparietal	Parietal			7.95	*	7.71	*						
35	Left-Cerebellum-Cortex	Cerebellum			4.28	**	4.04	**	3.73	***	-3.49	**		
36	Left-Thalamus-Proper	Sub-cortical GM			4.21	*	4.47	*						
37	Left-Caudate	Sub-cortical GM			4.33	***			2.00	*				
38	Left-Putamen	Sub-cortical GM					4.31	*						
39	Left-Pallidum	Sub-cortical GM			4.15	**	4.48	**	2.96	**	-3.30	**		
42	Left-Accumbens-area	Sub-cortical GM			-2.71	**								
44	Right-Caudate	Sub-cortical GM			3.46	*			2.14	*				
46	Right-Pallidum	Sub-cortical GM			3.41	*			2.58	*				
47	Right-Hippocampus	Sub-cortical GM												
48	Right-Amygdala	Sub-cortical GM			-2.41	**			-1.32	*				
50	Right bankssts	Temporal			-9.14	**			-8.54	***				
55	Right fusiform	Temporal			-7.14	*								
56	Right inferiorparietal	Parietal			-9.52	**			-7.69	**				
58	Right isthmuscingulate	Cingulate			2.80	*								
59	Right lateraloccipital	Occipital			-13.55	**			-13.22	***				
60	Right lateralorbitofrontal	Frontal							-3.50	**				
63	Right middletemporal	Temporal			-8.16	**			-6.99	***			-5.25	*
65	Right paracentral	Frontal			6.90	**	6.64	*						
67	Right parsorbitalis	Frontal			-5.71	*			-6.10	***				
69	Right pericalcarine	Occipital			-11.07	*			-9.34	*				
73	Right precuneus	Parietal			6.56	*			4.84	*				
82	Right transversetemporal	Temporal			-4.19	*			-3.50	**				
84	Right-Cerebellum-Cortex	Cerebellum			3.60	*	4.08	**	2.62	*	-3.10	*		

* $p<0.05$; ** $p<0.01$; *** $p<0.001$

Table S12. Detailed results for the weighted degree. Values represent differences between each pair of MS clinical forms (CIS vs RR, CIS vs SP, CIS vs PP, RR vs SP, PP vs RR, PP vs SP). Statistical significance is also reported.

			CIS vs RR	CIS vs SP	CIS vs PP	RR vs SP	PP vs RR	PP vs SP
1	Left bankssts	Temporal				396.68 *		
3	Left caudalmiddlefrontal	Frontal		1252.71 *	1211.53 *			
4	Left cuneus	Occipital		1168.55 **		1100.92 ***	-673.05 *	
8	Left inferiortemporal	Temporal				775.78 **		
9	Left isthmuscingulate	Cingulate		590.57 **	493.88 *	464.47 **	-367.78 *	
10	Left lateraloccipital	Occipital		1662.62 **	1833.78 **	1091.88 *	-1263.03 *	
12	Left lingual	Occipital		1053.87 **		874.81 ***		
15	Left parahippocampal	Temporal				191.59 **		
16	Left paracentral	Frontal		955.20 **		1086.58 ***		
20	Left pericalcarine	Occipital		1119.51 **	852.53 *	737.41 **		
21	Left postcentral	Parietal						
24	Left precuneus	Parietal		2281.14 ***	1793.58 **	1602.24 ***	-1114.68 *	
27	Left superiorfrontal	Frontal						
28	Left superiorparietal	Parietal		3166.28 **	2676.84 *	2386.99 **		
29	Left superiortemporal	Temporal						
33	Left transversetemporal	Temporal		202.32 **		159.80 **		
35	Left-Cerebellum-Cortex	Cerebellum				1165.62 *		
36	Left-Thalamus-Proper	Sub-cortical GM		1773.43 ***	1624.52 ***	1387.03 ***	-1238.12 ***	
37	Left-Caudate	Sub-cortical GM	506.20 *	1175.63 ***	910.54 ***	669.43 ***		
38	Left-Putamen	Sub-cortical GM		1308.96 **	1497.41 **	892.06 *	-1080.51 **	
39	Left-Pallidum	Sub-cortical GM		843.07 ***	884.09 ***	601.04 ***	-642.07 ***	
40	Left-Hippocampus	Sub-cortical GM				339.51 *		
43	Right-Thalamus-Proper	Sub-cortical GM		1671.28 ***	1684.08 ***	994.63 **	-1007.43 *	
44	Right-Caudate	Sub-cortical GM		1042.56 ***	743.73 *	466.53 *		
45	Right-Putamen	Sub-cortical GM		1340.31 **	1405.47 **			
46	Right-Pallidum	Sub-cortical GM		802.49 **	833.31 **	531.87 **	-562.70 **	
47	Right-Hippocampus	Sub-cortical GM		487.31 *	600.61 **	476.62 **	-589.91 ***	
48	Right-Amygdala	Sub-cortical GM		-210.82 **				
53	Right cuneus	Occipital		867.85 *		955.03 ***		
58	Right isthmuscingulate	Cingulate		615.63 **	551.49 **	497.02 ***	-432.88 **	
59	Right lateraloccipital	Occipital						
61	Right lingual	Occipital		923.08 *		817.93 **		
64	Right parahippocampal	Temporal				228.53 *		
65	Right paracentral	Frontal		1341.13 ***	1188.59 **	820.67 **		
69	Right pericalcarine	Occipital		1349.62 **		1247.47 ***		
70	Right postcentral	Parietal				967.96 *		
73	Right precuneus	Parietal		2099.80 ***	1365.96 *	1896.22 ***	-1162.39 *	
74	Right rostralanteriorcingulate	Cingulate				-191.16 *		
77	Right superiorparietal	Parietal		3402.80 **		2473.04 **		
84	Right-Cerebellum-Cortex	Cerebellum						

* $p<0.05$; ** $p<0.01$; *** $p<0.001$

Table S13. Detailed results for the weighted local efficiency. Values represent differences between each pair of MS clinical forms (CIS vs RR, CIS vs SP, CIS vs PP, RR vs SP, PP vs RR, PP vs SP). Statistical significance is also reported.

			CIS vs RR	CIS vs SP	CIS vs PP	RR vs SP	PP vs RR	PP vs SP
1	Left bankssts	Temporal				-6.89 *		
6	Left fusiform	Temporal						
8	Left inferiortemporal	Temporal						
9	Left isthmuscingulate	Cingulate		4.65 *				
10	Left lateraloccipital	Occipital				-10.22 *		
14	Left middletemporal	Temporal		-12.51 *		-9.99 *		
16	Left paracentral	Frontal		11.92 **		9.77 ***		
24	Left precuneus	Parietal		18.97 ***	14.51 *	13.48 ***		
27	Left superiorfrontal	Frontal		18.43 *		11.80 *		
28	Left superiorparietal	Parietal		17.28 *	16.77 *			
35	Left-Cerebellum-Cortex	Cerebellum		8.20 **	7.64 *	7.57 ***	-7.01 **	
36	Left-Thalamus-Proper	Sub-cortical GM			9.68 *	6.47 *	-7.72 *	
37	Left-Caudate	Sub-cortical GM		8.86 ***	5.89 *	4.44 **		
38	Left-Putamen	Sub-cortical GM			9.52 *		-6.79 *	
39	Left-Pallidum	Sub-cortical GM		7.36 **	8.67 **	5.54 **	-6.85 **	
42	Left-Accumbens-area	Sub-cortical GM		-2.88 *				
44	Right-Caudate	Sub-cortical GM		6.75 **				
46	Right-Pallidum	Sub-cortical GM						
47	Right-Hippocampus	Sub-cortical GM					-5.42 *	
48	Right-Amygdala	Sub-cortical GM		-4.65 ***	-3.28 *	-2.35 *		
50	Right bankssts	Temporal		-11.47 **		-10.41 ***		
55	Right fusiform	Temporal		-13.40 **				
56	Right inferiorparietal	Parietal		-14.36 *		-12.45 **		
57	Right inferiortemporal	Temporal		-9.18 *		-6.35 *		
58	Right isthmuscingulate	Cingulate		5.33 *				
59	Right lateraloccipital	Occipital		-19.72 *		-20.89 ***		
60	Right lateralorbitofrontal	Frontal				-5.08 *		
63	Right middletemporal	Temporal		-13.29 ***		-11.33 ***		-8.63 *
65	Right paracentral	Frontal		12.68 **	13.67 **			
67	Right parsorbitalis	Frontal		-6.70 *		-7.00 **		
73	Right precuneus	Parietal		14.43 **		11.71 **		
76	Right superiorfrontal	Frontal						
77	Right superiorparietal	Parietal		17.51 *				
79	Right supramarginal	Parietal				-14.37 **		
81	Right temporalpole	Temporal						
82	Right transversetemporal	Temporal				-2.65 *		
84	Right-Cerebellum-Cortex	Cerebellum		6.05 *	6.94 *	5.47 **	-6.36 **	

* $p<0.05$; ** $p<0.01$; *** $p<0.001$
